# *CD44* Gene Polymorphisms as a Risk Factor for Susceptibility and Their Effect on the Clinicopathological Characteristics of Lung Adenocarcinoma in Male Patients

**DOI:** 10.3390/ijerph17092981

**Published:** 2020-04-25

**Authors:** Ju-Pi Li, Ming-Ju Hsieh, Ying-Erh Chou, Yu-Hua Chao, Thomas Chang-Yao Tsao, Shun-Fa Yang

**Affiliations:** 1 School of Medicine, Chung Shan Medical University, Taichung 402, Taiwan; d888203@gmail.com (J.-P.L.); intointo814@gmail.com (Y.-E.C.); nka6150@gmail.com (Y.-H.C.); 2 Department of Pediatrics, Chung Shan Medical University Hospital, Taichung 402, Taiwan; 3 Institute of Medicine, Chung Shan Medical University, Taichung 402, Taiwan; 170780@cch.org.tw; 4 Cancer Research Center, Changhua Christian Hospital, Changhua 500, Taiwan; 5 Graduate Institute of Biomedical Sciences, China Medical University, Taichung 404, Taiwan; 6 Department of Medical Research, Chung Shan Medical University Hospital, Taichung 402, Taiwan; 7 Division of Chest, Department of Internal Medicine, Chung Shan Medical University Hospital, Taichung 402, Taiwan

**Keywords:** lung cancer, CD44, polymorphism, EGFR mutation

## Abstract

Lung adenocarcinoma is a subtype of lung cancer with high morbidity and mortality. CD44 is instrumental in many physiological and tumor pathological processes. The expression of unique single nucleotide polymorphisms (SNPs) contributes to protein dysfunction and influences cancer susceptibility. In the current study, we investigated the relationship between *CD44* polymorphisms and the susceptibility to lung adenocarcinoma with or without epidermal growth factor receptor (EGFR) gene mutations. This study included 279 patients with lung adenocarcinoma. In total, six *CD44* SNPs (rs1425802, rs11821102, rs10836347, rs13347, rs187115, and rs713330) were genotyped using a real-time polymerase chain reaction. We found no significant differences in genotype distribution of *CD44* polymorphisms between EGFR wild-type and EGFR mutation type in patients with lung adenocarcinoma. We observed a strong association between *CD44* rs11821102 G/A polymorphism and EGFR L858R mutation (odds ratio (OR) = 3.846, 95% confidence interval (CI) = 1.018–14.538; *p* = 0.037) compared with the EGFR wild-type group. In the subgroup of male patients with lung adenocarcinoma harboring the EGFR wild-type, both *CD44* rs713330 T/C (OR = 4.317, 95% CI = 1.029–18.115; *p* = 0.035) and rs10836347 C/T polymorphisms (OR = 9.391, 95% CI = 1.061–83.136; *p* = 0.019) exhibited significant associations with tumor size and invasion. Data from the present study suggest that *CD44* SNPs may help to predict cancer susceptibility and tumor growth in male patients with lung adenocarcinoma.

## 1. Introduction

Lung cancer is the leading cause of cancer-related death worldwide, and its morbidity and mortality are increasing [1]. Each year, non-small cell lung cancer (NSCLC) accounts for approximately 85% of newly diagnosed lung cancer cases, and adenocarcinoma, a subtype of NSCLC, accounts for approximately 50% of all lung cancers. Many risk factors, such as genetic, behavioral, and environmental factors, have been shown to be involved in the development of lung cancer. For example, studies of lung adenocarcinoma have revealed that epidermal growth factor receptor (EGFR) gene mutations occur more frequently in Asian populations than in Caucasians [2]. Among several *EGFR* mutations, two hotspot mutations, namely L858R and Exon 19-del mutations, comprise of the vast majority in patients with lung cancer [3]. Single nucleotide polymorphisms (SNPs) are the most common type of genetic variation [4]. The expression of SNPs reportedly contributes to protein dysfunction and has an effect on disease susceptibility [5]. Therefore, identification of gene polymorphisms provides useful clinical information for the prediction and diagnosis of certain diseases, including lung cancer.

Human *CD44* is located on chromosomal locus 11p13 and contains 20 exons, including 10 constant exons and 10 variant exons [6,7]. Through alternative splicing and post-translational modifications of mRNA, various CD44 protein isoforms are generated, including the standard form and spliced variant isoforms [8]. Each of the CD44 isoforms has multiple biological functions in normal and tumor cells. Transmembrane glycoprotein CD44 participates in many physiological processes, such as cell adhesion, migration, and inflammation. Additionally, CD44 proteins are involved in tumor pathological processes, including cell proliferation, angiogenesis, invasion, and metastasis [9]. Overexpression of CD44 proteins may be a poor prognostic indicator for patients with NSCLC [10]. In fact, both CD44 mRNA [11] and protein [12] are highly expressed in the tumor tissues of patients with NSCLC. Moreover, the expression of CD44 isoforms is reportedly highly correlated with the production of various tumor subtypes and has been used as a marker for cancer stem cells in many cancers [13]. However, whether *CD44* genetic polymorphisms affect the subtype and the content of these CD44 isoforms in various cancers is worthy of further research.

Many studies have indicated that *CD44* polymorphisms are a risk factor for susceptibility to different cancers [14,15,16,17,18,19,20]. However, the role of *CD44* polymorphisms in the clinicopathological characteristics of lung adenocarcinoma is still unclear. In the present study, we aim to clarify the relationship between *CD44* polymorphisms and susceptibility to lung adenocarcinoma with or without *EGFR* mutations. The associations among *CD44* SNPs, EGFR mutations, and the clinicopathological characteristics of lung adenocarcinoma are also evaluated.

## 2. Materials and Methods 

### 2.1. Subjects and Specimen Collection

This study included 279 patients with lung adenocarcinoma at Cheng-Ching General Hospital, Taiwan, between 2012 and 2015. Tumor specimens were collected for EGFR gene sequencing, and whole-blood specimens were collected for CD44 genotyping. Whole-blood specimens were collected from patients using EDTA-containing tubes. The clinical data of the enrolled patients was obtained from their medical records. The tumor was staged according to the tumor/node/metastasis (TNM) staging system of the American Joint Committee on Cancer (AJCC) at the time of diagnosis, and lifestyle variables (such as cigarette smoking) were collected using questionnaires. The study protocol was approved by the Institutional Review Board of Cheng-Ching General Hospital (No. HP120009; 22 September 2012). Signed informed consent was obtained from each participate before the initiation of the study.

### 2.2. Selection of CD44 Polymorphisms

In this study, 6 SNPs in the CD44 genome region were selected from International HapMap Project data. One SNP (rs1425802) in the promoter region and 3 SNPs (rs11821102, rs10836347, and rs13347) in the 3’ untranslated region (3’ UTR) of *CD44* were selected. In addition, 2 SNPs (rs187115 and rs713330) were selected because they have been associated with several cancers in Chinese Han populations [15,16,17,18].

### 2.3. Genomic DNA Extraction and Real-Time Polymerase Chain Reaction

Genomic DNA was extracted from whole-blood specimens of patients with lung adenocarcinoma using the QIAamp DNA Tissue kit and the QIAamp DNA Blood Mini Kit (Qiagen, Valencia, CA, USA), respectively, according to the manufacturer’s protocols. After preparing the DNA, it was aliquoted and stored at −20 °C and used as templates for the following experiments. Exons 18–21 of EGFR were amplified using a polymer chain reaction (PCR) and then subjected to DNA sequencing as described previously [21]. The allelic identification of 6 *CD44* SNPs (rs1425802, rs11821102, rs10836347, rs13347, rs187115, and rs713330) was examined using the TaqMan SNP genotyping assay and the ABI StepOnePlus Real-Time PCR system (Applied Biosystems, Foster City, CA, USA).

### 2.4. Statistical Analysis

The Mann–Whitney U test and Fisher’s exact test were used to compare differences in the distributions of clinical characteristics and genotype frequencies between lung adenocarcinoma patients harboring the EGFR wild-type and EGFR mutation type. The odds ratios (ORs) with corresponding 95% confidence intervals (CIs) of the association between the genotype frequencies and risk of EGFR types were estimated using multiple logistic regression models after controlling for other variables. The *p*-value less than 0.05 was indicated statistical significant. All data were analyzed using SAS (version 9.1, 2005; SAS Institute Inc., Cary, NC, USA).

## 3. Results

To explore the effects of CD44 polymorphisms on lung adenocarcinoma risk, a total of 279 whole-blood specimens were collected from lung adenocarcinoma patients. EGFR sequencing data were used to divide these specimens into the EGFR wild-type (*n* = 110, 39.4%) and EGFR mutation type (*n* = 169, 60.6%). The clinical characteristics of enrolled patients are presented in Table 1. We observed significant differences when grouping patients by sex (*p* < 0.001), cigarette smoking (*p* < 0.001), and cell differentiation (*p* = 0.001) in the patients with lung adenocarcinoma with the EGFR wild-type and those with the EGFR mutation type (Table 1). In comparison with the EGFR wild-type group, the EGFR mutation type group had a higher proportion of female patients (*n* = 109, 64.5%), nonsmokers (*n* = 131, 77.5%), well-differentiated tumors (*n* = 21, 12.4%), and moderately differentiated tumors (*n* = 138, 81.7%; Table 1).

The genotype distributions and associations between CD44 polymorphisms and lung adenocarcinoma are listed in Table 2. In the recruited lung adenocarcinoma patients, the alleles with the highest distribution frequency for rs1425802, rs187115, rs713330, rs11821102, rs10836347, and rs13347 were heterozygous for A/G, homozygous for A/A, homozygous for T/T, homozygous for G/G, homozygous for C/C, and heterozygous for C/T, respectively. To minimize the interference of confounding variables, adjusted odds ratios with 95% confidence intervals (CIs) were estimated using multiple logistic regression models after adjustment for variance. No significant differences were observed in the genotype distributions of CD44 polymorphisms between the EGFR wild-type and EGFR mutation type in the patients with lung adenocarcinoma (Table 2). In addition, there were no significant associations between CD44 polymorphism and the cancer susceptibility in terms of sex (Table A1), smoking manner (Table A2), and cell differentiation (Table A3).

Due to the different sex distribution of EGFR status in patients with lung adenocarcinoma in Taiwan, the association between CD44 polymorphisms and two EGFR hotspot mutations (L858R and exon19 in-frame deletion) in male patients was investigated. In comparison with the EGFR wild-type group, an association was observed between CD44 rs11821102 G/A polymorphism and EGFR L858R mutation (AOR = 3.991, 95% CI = 1.002–16.499; *p* < 0.05) but not for EGFR exon19 in-frame deletion (Table 3). No significant association was found between the other five CD44 polymorphisms and the two EGFR hotspot mutations in male patients (Table 3). In addition, no significant association was noted between CD44 polymorphisms and the 2 EGFR hotspot mutations in female patients (data not shown). The data suggested that men carrying at least one A allele in CD44 rs11821102 had a higher risk of developing lung adenocarcinoma harboring the EGFR L858R mutation than those carrying homozygous GG alleles.

Subsequently, we investigated the relationships between *CD44* polymorphisms and clinicopathological characteristics of male patients with lung adenocarcinoma (*n* = 126). Using the AJCC TNM staging system for lung cancer [22], we observed that the *CD44* rs713330 T/C polymorphism was highly associated with the “T” classification (AOR = 4.250, 95% CI = 1.529–11.814; *p* = 0.006) in male patients with lung adenocarcinoma (Table 4). To further examine the association between *CD44* polymorphisms and clinicopathological characteristics of EGFR status, these male patients were stratified into EGFR wild-type (*n* = 66) and EGFR mutation type (*n* = 60) groups. In the *EGFR* wild-type group, both *CD44* rs713330 T/C (AOR = 5.398, 95% CI = 1.203–24.223; *p* = 0.028) and rs10836347 C/T polymorphisms (AOR = 9.136, 95% CI = 1.028–81.200; *p* = 0.047) exhibited significant associations with the AJCC “T” classification (Table 4). These findings indicated that the *CD44* rs713330 T/C polymorphism might be associated with tumor size and invasion in male patients with lung adenocarcinoma, particularly those with wild-type *EGFR*.

## 4. Discussion

In the present study, we demonstrated the clinical relevance of *CD44* polymorphisms in male patients with lung adenocarcinoma. We revealed that these patients with the *CD44* rs11821102-variant genotypes (GA + AA) exhibited a higher risk of lung adenocarcinoma with the development of the *EGFR* L858R mutation. Furthermore, the *CD44* rs713330 T/C polymorphism exhibited a significant association with the AJCC “T” stage classification. The classification of the “T” stage depends on the size of the primary tumor and its invasion of adjacent tissues [22]. Therefore, the *CD44* rs713330 polymorphism may be associated with the primary tumor size and invasion of lung adenocarcinoma in male patients, particularly those with wild-type *EGFR*.

*EGFR* mutations have been found to predominantly occur in patients with lung adenocarcinoma and are more frequent in female patients and nonsmokers [2,23,24,25]. Most of the recruited patients with lung adenocarcinoma in the present study had *EGFR* mutation. Data indicated that *EGFR* mutations were more common in female patients than in their male counterparts, more common in those that had never smoked than in those who do smoke, and more common in well and moderately differentiated lung tumors in the collected samples. In addition, EGFR mutations are the prevalence of EGFR mutations in Asian populations is higher than that in Caucasians [2]. Indeed, the different sex distribution of the EGFR status in patients with lung adenocarcinoma is found in Taiwan. Since our study only explores the Taiwanese group, it needs to further study whether the CD44 gene polymorphism is as a risk factor for the susceptibility of male lung adenocarcinoma, particularly those with wild-type EGFR, in other races. Here we found that a strong association was observed between CD44 rs11821102 G/A polymorphism and EGFR L858R mutation in male patients, but not in female patients. The present data showed that sexual dimorphism was observed in the association between CD44 polymorphisms and the susceptibility of lung adenocarcinoma. Sex difference has been observed in most human diseases, including lung cancer, colorectal cancer, and malignant melanoma [21,26,27]. Previous studies have shown that polymorphisms in CYP1A1 and GSTM1 [28] and EGFR [21] contribute to the increased risk of females for lung cancer. Our data and these previous studies suggest that the genetic and hormonal diversity between men and women could affect the gene expression patterns, leading to different cancer progression and risks in the specific subgroup.

Human *CD44* encodes many protein isoforms. These CD44 isoforms have been reported to participate in many biological processes [9,13]. Here we demonstrated that male individuals carrying at least one A allele in *CD44* rs11821102 had a higher risk of lung adenocarcinoma harboring the *EGFR* L858R mutation than those carrying homozygous GG alleles. Rs11821102 is located in the noncoding *CD44* 3’ UTR, which affects certain microRNA binding capabilities and then interferes with mRNA expression [29]. The data indicated that the *CD44* rs11821102 G/A polymorphism might influence unique microRNA, thereby altering specific CD44 mRNA subtypes in male patients with lung adenocarcinoma harboring the *EGFR* L858R mutation. Future research could consider which microRNA is affected by *CD44* rs11821102 G/A polymorphism and investigate the underlying mechanisms.

Studies have been revealed that overexpression of the CD44 variant exon 6 is associated with tumor differentiation, clinical TNM stage, and lymph node metastasis in patients with NSCLC [10,30]. The expression of CD44 proteins in tumor tissues and serum samples from NSCLC has been found to be significantly associated with clinicopathological factors including T stage, N stage, and pathological stage [12,31]. We observed that the *CD44* rs713330 T/C polymorphism was highly associated with primary tumor size and invasion (AJCC “T” classification) in male patients with lung adenocarcinoma. Rs713330, located in the intron of *CD44*, is linked to the disequilibrium of the nonsynonymous rs9666607 G/A polymorphism, which may change arginine to lysine at residue 417 [15]. The present data suggest that the *CD44* rs713330 T/C polymorphism may change the CD44 protein subtype expression to be associated with tumor size and invasion in male patients with lung adenocarcinoma, particularly those with wild-type *EGFR*. Furthermore, in the subgroup of male patients harboring *EGFR* wild-type, the rs10836347 C/T polymorphism was also associated with tumor size and invasion. *CD44* polymorphisms may cause changes in the subtype and content of the mRNA and/or protein of *CD44*. Therefore, whether *CD44* genetic polymorphisms, such as rs713330 and rs10836347, directly affect the clinicopathological characteristics of lung adenocarcinoma merits further attention.

Several SNPs of CD44 have been reported to have significant correlations with the risk of various cancers in Asians [20,32]. The rs10836347 C/T polymorphism is associated with a higher risk of hepatocellular carcinoma in individuals who consume alcohol [33]. To our knowledge, this is the first report indicating that rs10836347 C/T and rs713330 T/C polymorphisms may increase lung adenocarcinoma risk and cause tumor growth, respectively, in male patients. Liu et al. revealed that rs187115 sites of the G allele significantly increase the risk of NSCLC and bone metastases compared with those of the AA genotype [34]. However, we did not observe the association between rs187115 and lung adenocarcinoma risk, which may be attributed to our lack of healthy controls and limited study samples.

Certain limitations still exist in the current study. Due to the lack of the overall survival and disease-free survival data, the impact between the CD44 polymorphism and follow up clinical data could not be performed. Moreover, it needs to conduct further research on larger cohorts, different races and even the international multicenters to investigate and confirm the association between these CD44 SNPs and clinicopathological characteristics patients with lung adenocarcinoma.

## 5. Conclusions

The present results suggest that CD44 rs11821102 G/A polymorphism is a potential candidate target for the prediction of lung adenocarcinoma with the EGFR L858R mutation in male patients. Our data enhance understanding of the involvement of CD44 SNPs in cancer susceptibility and clinicopathological characteristics in male patients with lung adenocarcinoma. Data from the present study suggest that CD44 SNPs may be a useful prediction marker for male patients with lung adenocarcinoma.

## Figures and Tables

**Table 1:** Demographics and clinical characteristics of 279 patients in lung adenocarcinoma with the epidermal growth factor receptor (EGFR) mutation status.

Variable	Wild Type (*N* = 110) *n* (%)	EGFR Mutation (*N* = 169) *n* (%)	*p* Value
**Age**			
< 65	53 (48.2%)	84 (49.7%)	*p* = 0.804
≥ 65	57 (51.8%)	85 (50.3%)	
Mean ± SD	65.36 ± 13.48	65.76 ± 13.57	*p* = 0.817
**Gender**			
Male	66 (60.0%)	60 (35.5%)	*p* < 0.001
Female	44 (40.0%)	109 (64.5%)	
**Cigarette Smoking Status**			
Never-smoker	49 (44.5%)	131 (77.5%)	*p* < 0.001
Ever-smoker	61 (55.5%)	38 (22.5%)	
**Stage**			
I + II	26 (23.4%)	47 (27.8%)	*p* = 0.438
III + IV	84 (76.4%)	122 (72.2%)	
**Tumor T Status**			
T1 + T2	60 (54.5%)	108 (63.9%)	*p* = 0.119
T3 + T4	50 (45.5%)	61 (36.1%)	
**Lymph Node Status**			
Negative	29 (26.4%)	54 (32.0%)	*p* = 0.318
Positive	81 (73.6%)	115 (68.0%)	
**Distant Metastasis**			
Negative	54 (49.1%)	80 (47.3%)	*p* = 0.774
Positive	56 (50.9%)	89 (52.7%)	
**Cell Differentiation**			
Well	8 (7.3%)	21 (12.4%)	*p* = 0.001
Moderately	80 (72.7%)	138 (81.7%)	
Poorly	22 (20.0%)	10 (5.9%)	

**Table 2:** Distribution frequency of *CD44* genotypes of patients with lung adenocarcinoma and multiple logistic regression analysis of EGFR mutation association.

	All Cases (*N* = 279)			
SNP Genotypes	Wild Type (*N* = 110)	Mutation Type (*N* = 169)	AOR (95% CI)	*p* Value
**rs1425802**				
AA	38 (34.5%)	64 (37.9%)	1.00	
AG	51 (46.4%)	77 (45.6%)	0.867 (0.499–1.507)	*p* = 0.613
GG	21 (19.1%)	28 (16.6%)	0.710 (0.345–1.462)	*p* = 0.353
AG + GG	72 (65.5%)	105 (62.1%)	0.821 (0.489–1.380)	*p* = 0.457
**rs187115**				
AA	74 (67.3%)	118 (69.8%)	1.00	
AG	33 (30.0%)	45 (26.6%)	0.846 (0.487–1.469)	*p* = 0.552
GG	3 (2.7%)	6 (3.6%)	1.438 (0.335–6.170)	*p* = 0.625
AG + GG	36 (32.7%)	51 (30.2%)	0.893 (0.524–1.520)	*p* = 0.676
**rs713330**				
TT	94 (85.5%)	147 (87.0%)	1.00	
TC	15 (13.6%)	21 (12.4%)	1.052 (0.502–2.207)	*p* = 0.893
CC	1 (0.9%)	1 (0.6%)	0.643 (0.036–11.491)	*p* = 0.764
TC + CC	16 (14.5%)	22 (13.0%)	1.024 (0.498–2.106)	*p* = 0.949
**rs11821102**				
GG	98 (89.1%)	149 (88.2%)	1.00	
GA	11 (10.0%)	17 (10.1%)	0.905 (0.396–2.070)	*p* = 0.813
AA	1 (0.9%)	3 (1.7%)	2.076 (0.199–21.675)	*p* = 0.542
GA + AA	12 (10.9%)	20 (11.8%)	0.995 (0.453–2.185)	*p* = 0.989
**rs10836347**				
CC	96 (87.3%)	141 (83.4%)	1.00	
CT	14 (12.7%)	27 (16.0%)	1.275 (0.623–2.607)	*p* = 0.506
TT	0 (0%)	1 (0.6%)	----	---
CT + TT	14 (12.7%)	28 (16.6%)	1.311 (0.643–2.673)	*p* = 0.456
**rs13347**				
CC	47 (42.7%)	76 (45.0%)	1.00	
CT	57 (51.8%)	79 (46.7%)	0.958 (0.571–1.609)	*p* = 0.873
TT	6 (5.5%)	14 (8.3%)	1.657 (0.578–4.749)	*p* = 0.348
CT + TT	63 (57.3%)	93 (55.0%)	1.025 (0.619–1.696)	*p* = 0.924

The adjusted odds ratios (AORs) with 95% confidence intervals (Cis) were estimated by multiple logistic regression models after controlling for age and gender. Abbreviations: SNP, single nucleotide polymorphism; AOR, adjusted odds ratio; CI, confidence interval.

**Table 3:** The associations between the polymorphisms of CD44 and the EGFR hotspot mutations in male patients with lung adenocarcinoma.

SNP Genotypes	Wild Type (*N* = 66)	L858R		Exon 19 in-Frame Deletion	
		(*N* = 18)	AOR (95% CI)	(*N* = 38)	AOR (95% CI)
**rs1425802**					
AA	23 (34.8%)	5 (27.8%)	1.00	19 (50.0%)	1.00
AG + GG	43 (65.2%)	13 (72.2%)	1.502 (0.462–4.889)	19 (50.0%)	0.567 (0.248–1.296)
**rs187115**					
AA	47 (71.2%)	12 (66.7%)	1.00	24 (63.2%)	1.00
AG + GG	19 (28.8%)	6 (33.3%)	1.283 (0.416–3.961)	14 (36.8%)	1.403 (0.592–3.323)
**rs713330**					
TT	55 (83.3%)	15 (83.3%)	1.00	32 (84.2%)	1.00
TC + CC	11 (16.7%)	3 (16.7%)	1.077 (0.256–4.529)	6 (15.8%)	1.159 (0.375–3.583)
**rs11821102**					
GG	60 (90.9%)	13 (72.2%)	1.00	37 (97.4%)	1.00
GA + AA	6 (9.1%)	5 (27.8%)	**3.991** (**1.002–16.499**)	1 (2.6%)	0.167 (0.019–1.496)
**rs10836347**					
CC	59 (89.4%)	17 (94.4%)	1.00	32 (84.2%)	1.00
CT + TT	7 (10.6%)	1 (5.6%)	0.488 (0.056–4.263)	6 (15.8%)	1.565 (0.474–5.166)
**rs13347**					
CC	24 (36.4%)	6 (33.3%)	1.00	16 (42.1%)	1.00
CT + TT	42 (63.6%)	12 (66.7%)	1.165 (0.386–3.519)	22 (57.9%)	0.795 (0.346–1.823)

Abbreviations: SNP, single nucleotide polymorphism; the AORs with 95% CIs were estimated by multiple logistic regression models after controlling for age. AOR, adjusted odds ratio; CI, confidence interval. Bold text indicated a significant association with *p*-value < 0.05.

**Table 4:** The associations between polymorphic genotypes of CD 44 rs713330 and rs10836347 and clinicopathological characteristics of male patients with lung adenocarcinoma.

Variable Genotypic Frequencies	Tumor AJCC “T” Classification			
	T1 + T2 *n* (%)	T3 + T4 *n* (%)	AOR (95% CI)	*p* Value
**CD44** (**rs713330**) **All Cases** (***n* = 126**)	(***n* = 72**)	(***n* = 54**)		
TT	65 (90.3%)	39 (72.2%)	1.00	
TC + CC	7 (9.7%)	15 (27.8%)	4.250 (1.529–11.814)	***p* = 0.006**
**CD44** (**rs713330**) **EGFR Wild Type** (***n* = 66**)	(***n* = 37**)	(***n* = 29**)		
TT	34 (91.9%)	21 (72.4%)	1.00	
TC + CC	3 (8.1%)	8 (27.6%)	5.398 (1.203–24.223)	***p* = 0.028**
**CD44** (**rs713330**) **EGFR Mutation Type** (***n* = 60**)	(***n* = 35**)	(***n* = 25**)		
TT	31 (88.6%)	18 (72.0%)	1.00	
TC + CC	4 (11.4%)	7 (28.0%)	3.490 (0.852–14.297)	*p* = 0.082
**CD44** (**rs10836347**) **All Cases** (***n* = 126**)	(***n* = 72**)	(***n* = 54**)		
CC	65 (90.3%)	44 (81.5%)	1.00	
CT + TT	7 (9.7%)	10 (18.5%)	2.194 (0.770–6.250)	*p* = 0.141
**CD44** (**rs10836347**) **EGFR Wild Type** (***n* = 66**)	(***n* = 37**)	(***n* = 29**)		
CC	36 (97.3%)	23 (79.3%)	1.00	
CT + TT	1 (2.7%)	6 (20.7%)	9.136 (1.028–81.200)	***p* = 0.047**
**CD44** (**rs10836347**) **EGFR Mutation Type** (***n* = 60**)	(***n* = 35**)	(***n* = 25**)		
CC	29 (82.9%)	21 (84.0%)	1.00	
CT + TT	6 (17.1%)	4 (16.0%)	1.023 (0.246–4.258)	*p* = 0.975

Note: the AORs with 95% CIs were estimated by multiple logistic regression models after controlling for age. AOR, adjusted odds ratio. CI, confidence interval. Bold text indicated a significant association with *p*-value < 0.05.

## Appendix A

**Table A1:** The associations between the polymorphisms of CD44 and the sex status in lung adenocarcinoma.

SNP Genotypes	All Cases (*n* = 279)			
	Male (*n* = 126)	Female (*n* = 153)	AOR (95% CI)	*p* Value
**rs1425802**				
AA	49 (38.9%)	53 (34.6%)	1.00	
AG	58 (46.0%)	70 (45.8%)	1.117 (0.663–1.883)	*p* = 0.678
GG	19 (15.1%)	30 (19.6%)	1.463 (0.729–2.935)	*p* = 0.284
AG + GG	77 (61.1%)	100 (65.4%)	1.201 (0.736–1.961)	*p* = 0.464
**rs187115**				
AA	86 (68.3%)	106 (69.3%)	1.00	
AG	35 (27.8%)	43 (28.1%)	0.997 (0.587–1.693)	*p* = 0.992
GG	5 (4.0%)	4 (2.6%)	0.647 (0.168–2.490)	*p* = 0.527
AG + GG	40 (31.7%)	47 (30.7%)	0.953 (0.573–1.585)	*p* = 0.854
**rs713330**				
TT	104 (82.5%)	137 (89.5%)	1.00	
TC	21 (16.7%)	15 (9.8%)	0.540 (0.265–1.101)	*p* = 0.090
CC	1 (0.8%)	1 (0.7%)	0.773 (0.047–12.632)	*p* = 0.857
TC + CC	22 (17.5%)	16 (10.5%)	0.551 (0.275–1.102)	*p* = 0.092
**rs11821102**				
GG	114 (90.5%)	133 (86.9%)	1.00	
GA	10 (7.9%)	18 (11.8%)	1.545 (0.685–3.487)	*p* = 0.294
AA	2 (1.6%)	2 (1.3%)	0.865 (0.118–6.338)	*p* = 0.886
GA + AA	12 (9.5%)	20 (13.1%)	1.435 (0.670–3.073)	*p* = 0.353
**rs10836347**				
CC	109 (86.5%)	128 (83.7%)	1.00	
CT	17 (13.5%)	24 (15.7%)	1.202 (0.614–2.354)	*p* = 0.591
TT	0 (0%)	1 (0.6%)	----	---
CT + TT	17 (13.5%)	25 (16.3%)	1.252 (0.643–2.440)	*p* = 0.509
**rs13347**				
CC	48 (38.1%)	75 (49.0%)	1.00	
CT	68 (54.0%)	68 (44.4%)	0.637 (0.388–1.047)	*p* = 0.075
TT	10 (7.9%)	10 (6.6%)	0.639 (0.247–1.650)	*p* = 0.355
CT + TT	78 (61.9%)	78 (51.0%)	0.638 (0.394–1.032)	*p* = 0.067

The AORs with 95% CIs were estimated by multiple logistic regression models after controlling for age. Abbreviations: SNP, single nucleotide polymorphism; AOR, adjusted odds ratio; CI, confidence interval.

**Table A2:** The associations between the polymorphisms of CD44 and the cigarette smoking status in lung adenocarcinoma.

SNP Genotypes	All Cases (*n* = 279)			
	Never-Smoker (*n* = 180)	Ever-Smoker (*n* = 99)	AOR (95% CI)	*p* Value
**rs1425802**				
AA	61 (33.9%)	41 (41.4%)	1.00	
AG	81 (45.0%)	47 (47.5%)	0.877 (0.408–1.885)	*p* = 0.736
GG	38 (21.1%)	11 (11.1%)	0.362 (0.129–1.017)	*p* = 0.054
AG + GG	119 (66.1%)	58 (58.6%)	0.699 (0.341–1.432)	*p* = 0.328
**rs187115**				
AA	123 (68.3%)	69 (69.7%)	1.00	
AG	53 (29.4%)	25 (25.3%)	0.713 (0.331–1.533)	*p* = 0.386
GG	4 (2.2%)	5 (5.1%)	2.938 (0.379–22.763)	*p* = 0.302
AG + GG	57 (31.7%)	30 (30.3%)	0.826 (0.396–1.722)	*p* = 0.611
**rs713330**				
TT	156 (86.7%)	85 (85.9%)	1.00	
TC	24 (13.3%)	12 (12.1%)	0.407 (0.156–1.061)	*p* = 0.066
CC	0 (0%)	2 (2.0%)	---	---
TC + CC	24 (13.3%)	14 (14.1%)	0.556 (0.220–1.405)	*p* = 0.214
**rs11821102**				
GG	157 (87.2%)	90 (90.9%)	1.00	
GA	21 (11.7%)	7 (7.1%)	0.626 (0.184–2.125)	*p* = 0.452
AA	2 (1.1%)	2 (2.0%)	2.355 (0.117–47.223)	*p* = 0.576
GA + AA	23 (12.8%)	9 (9.1%)	0.749 (0.239–2.352)	*p* = 0.621
**rs10836347**				
CC	153 (85.0%)	84 (84.8%)	1.00	
CT	26 (14.4%)	15 (15.2%)	1.467 (0.538–4.004)	*p* = 0.454
TT	1 (0.6%)	0 (0%)	----	---
CT + TT	27 (15.0%)	15 (15.2%)	1.448 (0.533–3.934)	*p* = 0.468
**rs13347**				
CC	83 (46.1%)	40 (40.4%)	1.00	
CT	83 (46.1%)	53 (53.5%)	0.925 (0.446–1.920)	*p* = 0.835
TT	14 (7.8%)	6 (6.1%)	0.454 (0.121–1.707)	*p* = 0.243
CT + TT	63 (53.9%)	59 (59.6%)	0.842 (0.415–1.707)	*p* = 0.633

The AORs with 95% CIs were estimated by multiple logistic regression models after controlling for age and gender. Abbreviations: SNP, single nucleotide polymorphism; AOR, adjusted odds ratio; CI, confidence interval.

**Table A3:** The associations between the polymorphisms of CD44 and the cell differentiation status in lung adenocarcinoma.

SNP Genotypes	All Cases (*n* = 279)			
	Well/Moderately-Differentiated (*n* = 247)	Poorly Cell Differentiated (*n* = 32)	AOR (95% CI)	*p* Value
**rs1425802**				
AA	90 (36.4%)	12 (37.5%)	1.00	
AG	115 (46.6%)	13 (40.6%)	0.886 (0.379–2.071)	*p* = 0.779
GG	42 (17.0%)	7 (21.9%)	1.487 (0.526–4.204)	*p* = 0.455
AG + GG	157 (63.6%)	20 (62.5%)	1.029 (0.471–2.246)	*p* = 0.943
**rs187115**				
AA	173 (70.0%)	19 (59.4%)	1.00	
AG	67 (27.1%)	11 (34.4%)	1.511 (0.671–3.402)	*p* = 0.319
GG	7 (2.9%)	2 (6.2%)	2.448 (0.450–13.325)	*p* = 0.300
AG + GG	74 (30.0%)	13 (40.6%)	1.605 (0.741–3.477)	*p* = 0.230
**rs713330**				
TT	215 (87.0%)	26 (81.3%)	1.00	
TC	31 (12.6%)	5 (15.6%)	1.158 (0.400–3.347)	*p* = 0.787
CC	1 (0.4%)	1 (3.1%)	8.266 (0.425–160.676)	*p* = 0.163
TC + CC	32 (13.0%)	6 (18.7%)	1.371 (0.509–3.696)	*p* = 0.533
**rs11821102**				
GG	220 (89.1%)	27 (84.4%)	1.00	
GA	23 (9.3%)	5 (15.6%)	2.048 (0.684–6.135)	*p* = 0.200
AA	4 (1.6%)	0 (0%)	---	---
GA + AA	27 (10.9%)	5 (15.6%)	1.677 (0.568–4.949)	*p* = 0.349
**rs10836347**				
CC	209 (84.6%)	28 (87.5%)	1.00	
CT	37 (15.0%)	4 (12.5%)	0.854 (0.278–2.630)	*p* = 0.784
TT	1 (0.4%)	0 (0%)	----	---
CT + TT	38 (15.4%)	4 (12.5%)	0.843 (0.274–2.594)	*p* = 0.766
**rs13347**				
CC	106 (42.9%)	17 (53.1%)	1.00	
CT	123 (49.8%)	13 (40.6%)	0.565 (0.256–1.248)	*p* = 0.158
TT	18 (7.3%)	2 (6.3%)	0.586 (0.121–2.848)	*p* = 0.508
CT + TT	141 (57.1%)	15 (46.9%)	0.567 (0.264–1.218)	*p* = 0.146

The AORs with 95% CIs were estimated by multiple logistic regression models after controlling for age and gender. Abbreviations: SNP, single nucleotide polymorphism; AOR, adjusted odds ratio; CI, confidence interval.
